# Management of Autistic Patients in Dental Office: A Clinical Update

**DOI:** 10.5005/jp-journals-10005-1515

**Published:** 2018-06-01

**Authors:** Shashidhar Chandrashekhar, Jyothi S Bommangoudar

**Affiliations:** 1 Professor, Department of Conservative Dentistry and Endodontics S.M.B.T. Dental College & Hospital, Ahmednagar, Maharashtra India; 2 Reader, Department of Pedodontics and Preventive Dentistry, S.M.B.T Dental College & Hospital, Ahmednagar, Maharashtra, India

**Keywords:** Autism, Behavioral approach, Cognitive and perceptional functioning, Oral health care.

## Abstract

Autism is an intellectual developmental disorder characterized by insidious disability in communication, social interaction, and using language and abstract concepts. This organic disorder is known to have deformities in brain, i.e., cerebellum and limbic system, showing wide spectrum of systemic and behavioral symptoms. The oral health care of such patients can be complicated as they cannot verbalize complaints about any dental problems they may be experiencing and can display a variety of behaviors and reactions to small changes also.

This study summarizes etiology and diagnosis of this disorder with the special emphasis on the issues encountered while coping with children with autistic spectrum.

**How to cite this article:** Chandrashekhar S, Bommangoudar JS. Management of Autistic Patients in Dental Office: A Clinical Update. Int J Clin Pediatr Dent 2018;11(3):219-227.

## INTRODUCTION

Autism is a complex neurodevelopment disarray defined as a behavioral syndrome, consisting of impaired social interaction, impaired communication skills (verbal as well as nonverbal language), sensorimotor deficits (unpredictable reactions to environmental stimuli) combined with stereotyped and restricted behaviors.^1^

Autistic patients constitute small percentage of special child population and they require unique management because of their behavioral characteristics.

### History

The word autism is derived from a Greek word “autos,” which means self, and “ismos,” which means a state of self-absorbed to the exclusion of everyone around them.^2^ The term autism is a name given to behavioral phenotype and was first coined by Bleuler in 1911, to denote a specific abandonment behavior disorder seen in schizophrenic patients.^3^

Kanner,^3^ an American psychiatrist, studied 11 children who have features like an extreme aloneness, lack of affective contact, difficulty in adapting any change in the routine, and increased sensitivity. Autistic disorder is categorized into the Diagnostic and Statistical Manual of Mental Disorders, 4th edition (DSM-IV) under the section, Pervasive Developmental Disorders (PDD), which is also referred to as autism spectrum disorder (ASD).

According to DSM-IV, PDD is an umbrella term used under which specific diagnoses are defined, which include autism disorder (AD), Asperger syndrome, rare disorders: Rett syndrome and childhood disintegrative disorder, and PDD not otherwise specified (or “atypical autism”). Individuals with ASDs will have difficulty in three domains: Social interaction, communication and repetitive behaviors, or restricted interests.^4^

### Etiology

The cause of ASD is not clear yet. Based on the available research, one can say that the disorder may be multi-factorial, involving genetics, environmental poisons, neuropsychopathy.

### Genetics

Recent research work has elucidated that parameters, such as CNTNAP2 gene, *de novo* mutations, mitochondrial defects,^5^ increased level of inflammatory cytokines, maternal bleeding during pregnancy, metabolic syndromes, and advancing maternal age^6^ are all persuasively linked to autism.

Neurological autism is an organic disorder associated with abnormalities in brain structure and function. Characteristic findings are a reduced number of Purkinje cells in the posterior-inferior regions of the cerebellar hemispheres, truncation in the dendritic tree development of neurons in the limbic system,^7^ and hypoplasia of cerebellar lobules VI and VII.^8^

Some patients with AD showed abnormal levels of serotonin or other neurotransmitters that can affect the brain development.^9^ Prenatal factors like intrauterine viral infections, or metabolic disorders and intrauterine exposures to the teratogenic drugs, thalidomide and valproate,^10^ may all play important role in the pathogenesis of AD.

Coexisting medical conditions of AD may be linked with seizure disorder, fragile-X syndrome, tuberous sclerosis, allergies, immune system problems, gastrointestinal disturbances, developmental delay, dysmorphic features, obstetric complications, and phenylketonuria.

### Incidence and Prevalence

Incidence of autism is about 0.2% in the US.^11^ Most recent study suggests the current prevalence rate as 1 in 110 children.^12^ The male:female ratio is 3.7:1,^13^ but females exhibit severe form of mental retardation. Higher prevalence rate in males suggests X-linked mode of inheritance.^14^

## CLINICAL FEATURES OF AUTISM

The onset of AD usually occurs before 3 years. There are some specific criteria referred by DSM for a patient to be diagnosed as AD, which include impairments in social functioning, deficits in communication, and restricted interests.^1^ The mean age noted for these deviations is 17 to 44 months.^15^ Early detection is necessary for early introduction of learning and behavioral guidance, which will give abiding benefits for these children and their families.^16^ Early signs and symptoms include baby does not seek parent attention, fails to cuddle, making direct eye contact, imitation games, no reaction to known faces, or other warm joyful expressions by 6 months of age,^17^ afraid of new things, try to make repeated actions to gain attention by 12 months of age, oversensitivity to textures, not able to understand pretend play by 16 months of age; 50% of patients do not develop speech. Abnormal and immature speech develops in virtue of intonation, pitch, rate, rhythm, grammar and word integration, understanding, and nonliteral speech.^1^

About 75% autistic patients suffer from moderate range of mental retardation.^18^ They don’t enjoy group activities and want to be in their own world and unable to share another child’s interest in an activity.^19^ Temper tantrums, hyperactivity, short attention span, impetuous, anxiety, anger, and a tendency for aggressive and self-injurious behaviors (SIBs) are common behavioral features in these patients. An automatic stereotyped behavior of psychogenic origin-like arm flapping and toe walking are common.

Echolalia and delayed echolalia are also present. Even small change in daily routine may increase or initiate it. This repetitive behavior is more frequent in females. The head and neck region is usually affected.^20^ The SIB can occur in 4 to 5% of individuals of autistic patients. It may be simple one, like self-pinching, or severe reactions, like self-biting or head banging, can even involve oral structure, such as lip biting and deep gingival cleft on canine caused by scrapping with finger nail.^21^ The cause is not clear. It might be to gain attention of caregivers or avoid unwanted events.

Understanding interpersonal dynamics will help in giving therapeutic approaches. Suggested therapeutic approaches consist of rewarding good conduct after completion of every step in a procedure, distracting patient from an undesired action, and inserting a prefabricated oral screen as a temporary physical distraction also helps diagnosis. Diagnosis of autism is completely based on observation of child’s behavior, psychosomatic testing, and history from parents, as there is no specific test or biomarker for autism.

Diagnosing AD involves developmental screening, followed by a comprehensive diagnostic evaluation. General developmental screening is performed for children to evaluate basic learning skills, such as behavior, speech, and movement are at appropriate time. Age intervals for this screening are 9, 18, 24, and 30 months. This screening specifically designed for autistic child are at the age of 18 and 24 months: Modified checklist for autism in toddlers (M-CHAT) and screening tool for autism in toddlers and young children (STAT).

After developmental screening, if any child is showing delay in attaining the developmental milestones, then comprehensive diagnostic evaluation is done to diagnose the AD. The evaluation is done by specialists, such as developmental pediatrician, psychologists, and child neurologists.

This evaluation involves thorough review of child behavior and development, interview with the parents, genetic and neurological tests of patients. The diagnostic criteria for autism (Table 1) is given by The National Autism Plan for Children.^22^

## PROGNOSIS

Development of speech is the best favorable outcome by 5 years.^23^ Autism disorder is a lifelong disorder, with no remission. The symptoms may change or lessen or disappear. The patients with low intelligence quotient (IQ) and low functioning need a protected environment throughout their life, whereas those patients with higher IQs will be able to live and can work with only minor supervision.

For medical line of treatment, psychotropic drugs, like antidepressants, antipsychotics, anticonvulsant, and central nervous system-stimulant drugs, are necessary to manage associated aggression, hyperactivity, self-mutilation, and peevishness.^24^ Larger doses of pyridoxine are the most widely used alternative medication.^25^ Many of these drugs cause systemic as well as oral side effects. It is essential for the dentists to know the properties of these drugs (Table 2).

**Table 1:** **Table 1:** Diagnostic criteria for autistic disorder (NAPC- the national autism plan for children)

A	A total of six items from the following criteria 1, 2, and 3, with at least two from criterion 1 and one each from criteria 2 and 3
1.	Qualitative impairment in social interaction
	a. Marked impairment in the use of multiple nonverbal behaviors such as eye-to-eye gaze, facial expression, body posture, and gestures to regulate social interaction
	b. Failure to develop peer relationships appropriate to developmental level
	c. Lack of spontaneous seeking to share enjoyment, interests, or achievements with other people (e.g., lack of showing, bringing, or pointing out objects of interest)
	d. Lack of social or emotional reciprocity.
2.	Qualitative impairments in communication
	a. Delay in or total lack of development of spoken language (not accompanied by an attempt to compensate through alternative modes of communication such as gesture or mime)
	b. In individuals with adequate speech, marked impairment in the ability to initiate or sustain a conversation.
	c. Stereotyped and repetitive use of language or idiosyncratic language
	d. Lack of varied, spontaneous make-believe play or social imitative play appropriate to developmental level.
3.	Restricted, repetitive, and stereotyped patterns of behavior, interests, and activities
	a. Encompassing preoccupation with one or more stereotyped and restricted patterns of interest that is abnormal either in intensity or focus
	b. Apparently inflexible adherence to specific, nonfunctional routines or rituals
	c. Stereotyped and repetitive motor mannerisms (e.g., hand or finger flapping or twisting, or complex whole-body movements
	d. Persistent preoccupation with parts of objects
B.	Delay or abnormal functioning in at least one of the following areas, with onset before age 3 years:
	a. Social interaction
	b. Language as used in social communication
	c. Symbolic or imaginative play
C.	Disturbance not better accounted for by Rett’s disorder or childhood disintegrative disorder

**Table 2:** **Table 2:** Side effects of drugs commonly used in the treatment of autism

*Drugs*		*Side effects*	
CNS stimulants (methylphenidate, dextroamphetamine salts)		Xerostomia	
Antipsychotics (Risperidone, Clozapine, Olanzepin, Quetiapine)		Xerostomia, sialorrhea, dysphagia, dysgeusia, stomatitis, gingivitis, tongue edema, glossitis, discolored tongue, difficulty in swallowing and orthostatic hypotension	
Antidepressants (Fluoxetine and Sertraline)		Xerostomia, sialadenitis, dysphagia, dysgeusia, stomatitis, gingivitis, glossitis, discolored tongue and bruxism	
Anticonvulsants (Carbamazepine and valproate)		Xerostomia, dysgeusia, stomatitis, glossitis. Prolonged therapy or medication combined with aspirin or non-steroidal anti-inflammatory drugs may result in excessive bleeding due to thrombocytopenia and leukopenia	
Antihypertensive ( clonidine)		Xerostomia, dysphagia, sialadenitis	

One of the commonly prescribed antidepressant, Fluoxetine, causes serious allergic reactions, like swelling of tongue, throat, and face, making prophylaxis difficult for both patient and dental hygienist. Local anesthesia with vasoconstrictor should not be given when methyl-phenidate is specially used for hyperactivity in autism as it may lead to tachycardia or a hypertensive episode.^26^

## ORAL HEALTH STATUS AND DENTAL CARIES

Institutionalized autistic individuals who had a higher frequency of and more serious periodontal problems, however, exhibited low caries.^27^ A low prevalence of dental caries was mentioned by Kamen and Skier.^28^ Lowe and Lindemann^29^ assessed the decayed, missing, filled teeth of autistic patients in primary dentition, which were high at the initial examination and were reduced on follow-up examinations, whereas Desai et al^30^ reported that dental caries rates were higher in autistic children of Melbourne, Australia. As children with autism have poor tongue coordination, they prefer soft and sweetened foods and tend to pouch food inside the mouth instead of swallowing it. Due to preference of food, long time presence of food within the oral cavity and difficulties in brushing and flossing due to lack of motor coordination and high sensitivity to taste of toothpaste, there is increased propensity to caries.^31^ Unique fixation of diet and preference for low-textured food by these autistic patients can contribute to low incidence of caries.^32^

Most of the times, tooth eruption might be delayed due to gingival hypertrophy which is caused by phenytoin.

Dental injuries were also common in autistic patients owing to the need of skills. These patients exhibit higher tendency to certain malocclusions, like ogival palate, crowding, and open bite. Harmful oral habits like nocturnal bruxism, tongue thrusting, and lip biting and gingival pricking were common.

## BARRIERS FOR ACCESS OF DENTAL TREATMENT

During the dental treatment, the main challenge is reduced ability of autistic kids to communicate and relate to others.^28^ Other problems like lack of capability to manage their emotions, repetitive body movements, hyperactivity associated with attention deficiency, and low frustration threshold can lead to peevishness and bizarre vocalizations.^21^ Sensations or sensory issues involve visual, auditory, olfactory, gustatory (taste and texture), and tactile cues.

The dental team should be organized for changeable and atypical responses to sensory stimuli, as these patients dislike even minute changes in their surroundings and require resemblance in continuity.^29^ Kopel^32^ reported that there will be high level of peripheral vision in autistic children. So lateral movements of any toy just before the patient are potential distractions should be avoided. These movements can change the behavior of child.^30^ Peripheral vision can be used by the autistic children to get more reliable information.^31^

## CLINICAL MANAGEMENT

Children with autism ought to have a patient-centered medical home that provides a constant, complete, family-centered, harmonized, empathetic, and culturally effective care, which can be easily reachable.

The objectives of management of autistic patients are to enhance independent performance, improve community engagement, and provide support to their parents and caregiver. A successful enduring management plan requires coordinating the efforts of educators, therapists, physicians, and mental health professionals.^33^

### Treatment Strategies

The role of family parental counseling: Previsit meeting is to prepare the family for first dental visit, which will be beneficial for both parents as well as dental team. Previsit meeting will help the parents in preparing the child for dental treatment and any hesitant problems related to child behavior should be discussed and must conquer.

The parents should understand that though their child has difficult time, the dental team support remains the same. Carefully listening to the parents/caretakers is a key element in gaining their trust, which in turn will help tremendously in gathering data.^21^ Dentist should know the medical conditions of the child and should be ready for co-occurring medical and physical issues.

Potential areas of concern during dental treatment for an ASD may worsen the symptoms, like use of fluoride, amalgam, and exposure to dental products with gluten and casein.^22^ Dentists should explain the parent and endeavor to give quality care without harming the child.

The major controversy about the fluoride application is possible neurotoxin, and two other possible effects are gastrointestinal irritation and dental fluorosis. So fluoride application in autistic patients is controversial, until unless the patient is under high risk for caries.^22^ The amalgam is durable restoration. When it comes to composites, it raises the health concern as it contains Bisphenol A, which is endocrine disrupter.

Generally, gluten-free, casein-free diet is recommended in autistic patients as these patients have a “leaky gut” where intestine is permeable to larger molecules like gluten from wheat, rye, and barley and casein from dairy products. These large molecules enter into bloodstream and tend to have negative effects on brain function and immune system.^22^

## DENTAL CLINIC ENVIRONMENT

The front desk receptionist is a key in helping patient’s future visits. For a successful dental visit of autistic child, the whole staff should be caring, empathetic, and aware of how to communicate with these patients.

Patients are likely to be disturbed emotionally by surrounding distracting stimuli like sound, light, and taste. Such discomfort may be reduced by adjusting the dental clinic environment sensitively.^34^ The experimental introduction of relight conditions, rhythmic music, and deep pressure in the dental setting diminished adverse patient reactions and enhanced active involvement in oral prophylactic procedures.^34^

It may be feasible to treat the patient in a calm, secured sole operatory with reduced decoration and dimmed lights.^21^ A single operating room may be also reserved to accommodate the treatment of the autistic child.

### Appointment Structure

The goal of the initial appointment is to establish trust and develop a relationship.^35^ It is important to learn what the patient is capable of doing *vs* learning what the patient is not able to do.^36^ Short,^37^ well-organized appointments should be planned and the waiting time should not exceed 10 to 15 minutes to avoid upset, as these patients have very limited attention span.^28^ To address the autistic individual’s preference for sameness and aversion to change, a routine should be established by maintaining days, times, and personnel for each dental visit.^38^ Discussions of any procedure of actual work should be avoided during its course.

Anyone who is involved in the management should reduce the movements because the autistic child is easily distracted.^39^ Pictures of dental clinic or a story can be given to parents to make acquaint the patient about clinic and reassure the comfort.^35^

### Dental Assistant Roles

Typically, it is the dental assistant or hygienist who will first contact with patient. Assistants should be able to identify triggering points of deviant reactions without fail.^40^

## BEHAVIOR GUIDANCE TECHNIQUES

Goals of behavior guidance are to develop rapport, lessen anxiety, and provide quality dental treatments while building a trusting and positive relationship for a lifetime between the professional and patient.^41^ However, higher rate of pliability is needed to meet the needs of quickly changing patient.^21^ Inappropriate behavior should be ignored. Hand over mouth is not considered an appropriate technique for these patients.^32^

### Communication

**Fig. 1:**
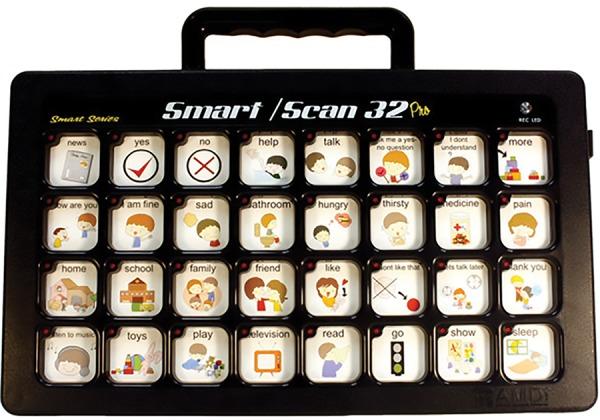
Smart/Scan 32 pro, an augmentative communication device

**Fig. 2:**
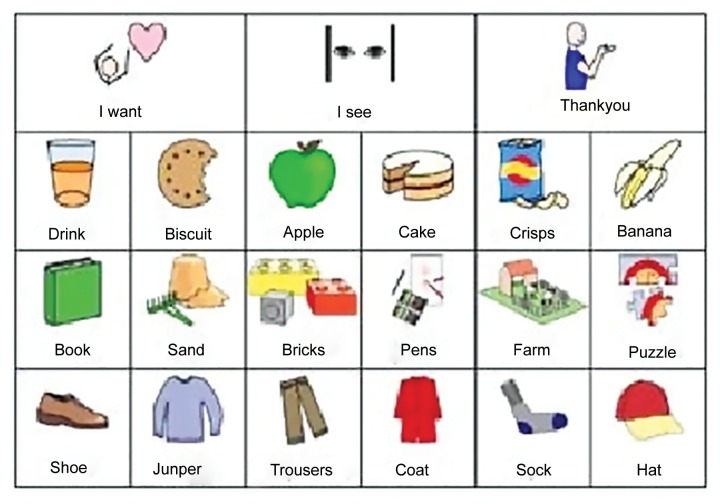
Picture exchange communication system

Communication guidance helps to establish trust and builds needed cooperation. Oral commands should be short, clear, and simple sentences. It is important to maintain good, ongoing communication throughout the visits and even after that. The ability to follow directions, learn new things, and articulate wants and needs may be difficult for some patients with autism. Some require assistive communicative devices, such as a Smart/Scan 32 pro, an augmentative communication device (Fig. 1), or a Picture Exchange Communication System (PECS) (Fig. 2). For autistic patients, PECS is an alternative communication technique with no or little verbal skills.

The PECS consists of a book of pictures to express desires, observations, and feelings. The book grows as the patient grows, with more words and pictures and is very helpful for those who are nonverbal.^36^

### Tell-Show-Do

“Tell-Show-Do” is a basic and effective exposure therapy and a way to introduce dental instruments, equipment, or procedures to a patient.^42^ For individuals with limited language, use pictures or objects to explain what will occur. Example: Pictures of radiographic film, disposable plastic mouth mirrors, mouth props or rests, saliva ejectors/ suction tips. Some individuals will benefit from practicing certain aspects of a procedure before experiencing them in a dental office.

### Restraints/Deep Pressure Touch

Concerning the use of restraints for autistic children, there is a controversy. Some authors advocate restraint board for calming down these patients in dental setting.^43^ But some disapprove. McDonald and Avery^31^ reported that restraints were used in challenged patients to obtain safer working conditions and more predictable working conditions. Various studies reported that applying a more or less firm wrap, pressure, and/or touch on emotionally disturbed or oversensitive persons can have a positive calming and comforting effect.^21^ Deep touch had a soothing effect, whereas light pat can be a tonic to the nervous system.^44^ One method that being wrapped in blankets or applying tight pressure to the entire body (e.g., a wet suit) lets them feel the limits of their body, and this has a soothing effect.^45^

### Desensitization

Desensitization techniques based on classical conditioning theory are required to diminish the apprehension present in autistic patients, which is very severe. Such techniques are time-consuming. Kopel^32^ suggested familiarizing the child with basic dental procedures in home. This technique involves dividing dental procedures into smaller steps. Each procedure should be successfully completed by a slow and step-wise approach and achievement of specific behavior. Then only next step is introduced.

### Voice Control

Before the use of this technique, the parents/caregiver should be well versed about the voice control, to avoid any misunderstandings during treatment. It can be used in any patients; however, autistic patients with hearing deficits would not be good candidates. Phrases, such as “eyes to me,” “look at me,” “hands on tummy,” or “feet straight out” can be used to elicit appropriate behaviors. If the patient is able to understand nonverbal communication, the use of nonverbal cues is a good way of eliciting appropriate behavior.^46^

### Positive Reinforcement

Positive reinforcement rewards preferred behaviors and thus strengthens the recurrence of the behavior. Oral admire and the pat of warmth, along with tokens of appreciation, can be used as positive reinforcers. The presence of the parent during the procedure is a good positive reinforcer. The parent presence is used to get the patient’s attention and increase compliance, decrease negative behaviors, establish appropriate roles during treatment, provide effective communication between dental provider and patient, and provide a positive dental experience.^46^

### Distractions

Distracting techniques like watching a favorite cartoon, listening to music, or holding onto special toys might help autistic patient to getting distracted while undergoing some procedures. Some techniques can engage the patient like holding balloon filled with water, an accordion tube. Autistic patients with high intellectual can be distracted by soothing and relaxed enough to undergo a procedure.^46^

### Sensory Techniques

It is essential to decrease the exposure of auditory and taste stimuli for autistic patients. During the dental visit, any drastic exposure to senses should be least,^37^ pertaining to oral hygiene, objectionable taste of toothpaste, and the sensation of toothbrush may hinder the effect of brushing.^47^ A gentle introduction to toothbrushing using alternatives, such as a washcloth, toothbrushes of different texture and design, or an electric toothbrush may enhance the acceptance of toothbrush by the child with ASD. Dentist or parent can be helpful by selecting the toothpaste with tolerable taste.

### Social Stories

Social stories help an individual to understand the events and what to look forward during the dental visit. During the previsit consultation with the family, functional behavioral evaluation can be done by the dentist.

**Fig. 3:**
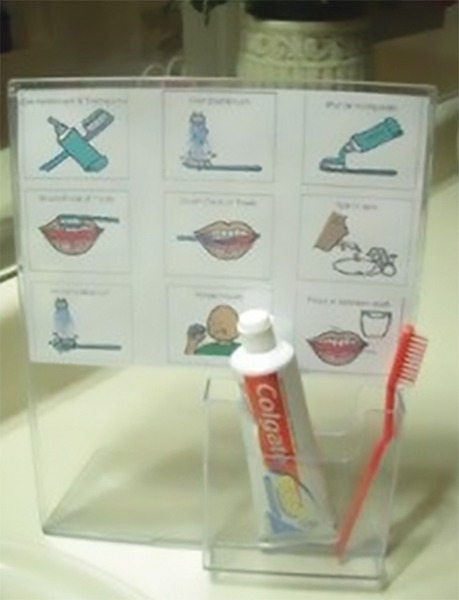
Visual pedagogy

During the previsit, one can introduce the dental instruments, teaching the skills of dental examination and tour into the clinic.^48^ Visual pedagogy (Fig. 3) involves the series of colored photographs describing step-by-step dental visit and toothbrushing to introduce oral hygiene to autistic children. Many autistic children are visual learners. Visual schedule may help to reduce the apprehension in children by understanding the sequence of procedures. Individuals come to know what steps have been completed and which are remaining.

## APPLIED BEHAVIOR ANALYSIS USING BEHAVIORAL LEARNING THEORIES

Applied behavior analysis (ABA) helps to change the behaviors by teaching specific skills. It functionally analyses the antecedents of behavior and consequences that follow.

In dentistry, the use of ABA practices has a high chance of improving the outcome of conventional behavior management techniques.^48^ By increasing the likelihood of patients who accept simple and routine dental procedures, dentists can decrease the need for more intrusive procedures, such as restraints and sedation.^33^ Each component of this skill would be divided into specific steps, each step would be taught separately, and a child would be rewarded as they learned each component skill. In shaping, the child is reinforced to adopt the behavior eventually on his own initiative.

With this method, the child can be trained to sit on the dental chair by themselves.^48^ Reinforcement represents one of the elemental concepts. It is considered to occur when there is an increase in certain behavior, as a consequence of a stimulus. The positive and negative reinforcements are linked to initiation and termination of the stimulus respectively.

For example, positive reinforce like rewarding with a toy or praising may lead to enhanced compliance in the dental chair. Conversely, negative reinforce like drilling can be handled by doing the procedure for predetermined period like counting from 1 to 10. Immediately after that, the procedure is interrupted for a while. The sequence of events is repeated as long as necessary for the procedure to be completed.^39^

### Aversive Techniques

The practice of hand-over-mouth exercise is rarely used these days because its use may be misconstrued as an assault.^49^ Although using physical restrainers in treating autistic child is also controversial, it may be applied effectively in some situations as a protectively supporting device for the patient, when used properly under informed consent.^50^ The use of restraints needs to determine according to each child.^45^

### Pharmacological Management

Conscious sedation had variable effect on autistic child. Usually, the physician is aware of an underlying health problem that would be a contraindication for sedation. If the patient has minimal dental treatment needs that can be accomplished in two operative appointments or less, then conscious sedation can be selected as a treatment plan. The sedation drugs most commonly used alone or in combination are: Versed, Vistaril, Demerol, Chloral Hydrate, and Nitrous Oxide.

During sedation, a patient must be monitored with a blood pressure and heart monitor, pulse oximeter, and a precordial stethoscope. There must be a second assistant employed to document these vital signs every 5 minutes during a sedation appointment. Some authors noted that administration of long duration and higher concentration of nitrous oxide than usual is required to get the preferred level of sedation in patients with AD.^42^ Management of autistic patients under general anesthesia is effective and it will help the patients to tolerate conventional treatment.

## CONCLUSION

As each patient is an individual, a thorough understanding about each patient is necessary for dentist and assistant. Simultaneously, parent should also have knowledge about the treatment given to their offspring is suitable and what is comfortable for him. Emotional skills will be more useful than intellectual and clinical skills.

The ability to handle the patients should be guided by instinct and creativity, rather than by strict reasoning. This article has projected on slight modifications of each behavior management techniques that are helpful for treating autistic patients. Over that, it is the flexibility and creativity of dentist and staff to give optimal care to the patients.
